# Effect of Circadian Clock and Light–Dark Cycles in *Onchidium reevesii*: Possible Implications for Long-Term Memory

**DOI:** 10.3390/genes10070488

**Published:** 2019-06-27

**Authors:** Guolyu Xu, Tiezhu Yang, Heding Shen

**Affiliations:** 1 National Demonstration Center for Experimental Fisheries Science Education, Shanghai Ocean University, Shanghai 201306, China; 2 International Research Center for Marine Biosciences, Shanghai Ocean University, Ministry of Science and Technology, Shanghai 201306, China; 3 Key Laboratory of Exploration and Utilization of Aquatic Genetic Resources, Shanghai Ocean University, Ministry of Education, Shanghai 201306, China

**Keywords:** long-term memory, light–dark cycle masking, circadian rhythm, *cry1*, *per2*, *OnCREB1*

## Abstract

The sea slug *Onchidium reevesii* inhabits the intertidal zone, which is characterized by a changeable environment. Although the circadian modulation of long-term memory (LTM) is well documented, the interaction of the circadian clock with light–dark masking in LTM of intertidal animals is not well understood. We characterized the LTM of *Onchidium* and tested the expression levels of related genes under a light–dark (LD) cycle and constant darkness (i.e., dark–dark, or DD) cycle. Results indicated that both learning behavior and LTM show differences between circadian time (CT) 10 and zeitgeber time (ZT) 10. In LD, the *cry1* gene expressed irregularly, and *per2* expression displayed a daily pattern and a peak expression level at ZT 18. *OnCREB1* (only in LD conditions) and *per2* transcripts cycled in phase with each other. In DD, the *cry1* gene had its peak expression at CT 10, and *per2* expressed its peak level at CT 18. *OnCREB1* had two peak expression levels at ZT 10 or ZT 18 which correspond to the time node of peaks in *cry1* and *per2,* respectively. The obtained results provide an LTM pattern that is different from other model species of the intertidal zone. We conclude that the daily transcriptional oscillations of *Onchidium* for LTM were affected by circadian rhythms and LD cycle masking.

## 1. Introduction

The sea slug *Onchidium reevesii* (Gastropoda: Eupulmonata: Systellommatophora) inhabits the intertidal zone in rocky, muddy habitats. The endemic mollusk forages on the reed bed during low tide and homes during high tide [1]. Many intertidal organisms such as *Onchidium* show more than one biological rhythm for their variable habits [2,3,4]. *Onchidium* is an organism with a rather small number of neurons in its central nervous system. Additionally, its central nervous system is visible and easily dissected [5]. 

The Earth’s ecosystem is governed by diverse environmental cycles, yet all are ultimately governed by the rotation of the Earth around the Sun. These cycles produce environmental changes in light, temperature, and so on. Different species harbor specific rhythms to take advantage of these variable environmental conditions and to avoid predators [6,7]. The most common cycle is the day–night cycle; thus, organisms need to subtly attune to this cycle and develop rhythms to conform to this steady 24 h, day and night pattern. In recent years, key components of the core oscillator mechanism have been identified [8,9,10]; one key gene of the circadian rhythm mechanism is period (*per*). The period protein, as a part of the circadian clock components, is thoroughly studied in insects and mammals [11,12,13]. Other common rhythms are the circatidal rhythm and circalunar rhythm; even though the circadian rhythm is well characterized, little is known about the circalunar rhythm and circatidal rhythm in marine invertebrate species. The tidal and lunar cycles robustly influence intertidal animals. Besides its role as a circadian clock gene, previous research suggested that cryptochrome (*cry*) is part of the tidal activity [10,14]. The *cry* is strongly related to UV-induced DNA damage repair and is sensitive to moonlight [15]. Some studies have shown that the lunar-dependent gene *cry* could also function as a state variable determining the lunar phase [14,16].

The circadian rhythm regulates a multitude of physiological activities [17] and also influences learning behaviors and long-term memory [18,19]. Long-term memory (LTM) is mediated by the *CREB* gene [20,21,22]; normally, more than one transcription factor plays an important role in both the signal path of circadian rhythms and in memory behaviors [23,24], and CREB1 is one of these transcription factors. Initial studies suggest that CREB1 activates the expression of downstream genes in the process of long-term memory in Aplysia [25,26], and the functional relationship between CREB activity and period gene expression has also been reported [27]. 

However, the regulatory effects of circadian rhythm on learning and memory have been investigated with different results. The most widely accepted result is that the time of day influences memory [19,28,29]. Some researchers found time-of-day effects on LTM [29]; however, some obtained contradictory results [30]. Interestingly, in some intertidal animals, light–dark cycle input could override the tendency of organisms to display a tidal rhythm of activity [3,31]; this is the result of the phenomenon called “masking” [32]. The key issue that has remained obscure is whether the light–dark (LD) cycle stimulates LTM directly in intertidal animals, or if the LD cycle independently influences their circadian rhythm and then induces LTM pattern change. 

This study aimed to determine the pattern of LTM rhythms and the oscillations of rhythm genes expressed by *Onchidium reevesii* when exposed to an LD cycle and complete darkness (i.e., dark–dark, or DD) cycle. In the behavioral experiments, the data obtained permitted us to test the hypothesis that the LD cycle influenced the animal’s activities. Learning abilities were subject to the cycle change; while the evidence showed that memory consolidation could be indirectly regulated by cycle change, as memory was determined by the amount of learning. The activity pattern of the mantle-upturned reflex in LD and DD expressed rhythmically and, to create enough data to determine the relationship between circadian rhythm and LTM in different cycles, we tested the expressions of the memory-related gene *OnCREB1*, circadian gene *per2*, and circalunar/circatidal-related gene *cry1* in different cycles after training. 

In the molecular experiments, we determined whether *per2* expressions were subject to circadian rhythms in LD and DD cycles after memory retrieval; moreover, we detected whether the *cry1* expressions remained rhythmic in LD and DD cycles. *Per2* expressed a daily rhythm in both LD and DD cycles, and *cry1* expressed rhythmically only in DD, suggesting that the LD cycle disturbs the rhythmicity of *cry1* expression. *OnCREB1* had the same expression pattern as *per2* in LD, and the peak expressions of *OnCREB1* are combinations of peak expressions of *per2* and *cry1* in DD, suggesting that LD cycles might influence LTM by disturbing other rhythms rather than the circadian rhythm. 

## 2. Materials and Methods

### 2.1. Experimental Animals

In all studies, we followed the guidelines of the Care and Use of Laboratory Animals issued by the Chinese Council on Animals Research and Guidelines of Animal Care. The study was approved by the ethical committee of Shanghai Ocean University (No. Shou-DW-2018-023). *Onchidium reevesii* (9–13 g) were reared in plastic tanks (25–28 °C) in Shanghai Ocean University for one month in good experimental conditions [33]. Animals were not used in experiments if they showed signs of infection or if they showed inactivity.

Animals were entrained to 12 h light–12 h dark (LD) cycles (total light intensity exceeded 100 lux during the light period and was under 6 lux during the dark period) for at least 10 days before training, and feeding was stopped one week before the training started.

Some of the animals were in the trained group, while others were in the native group without any training. Animals in the native group were also divided into two conditions. The native animals without any training were dissected at the same time as those in the trained group. 

**Figure 1:**
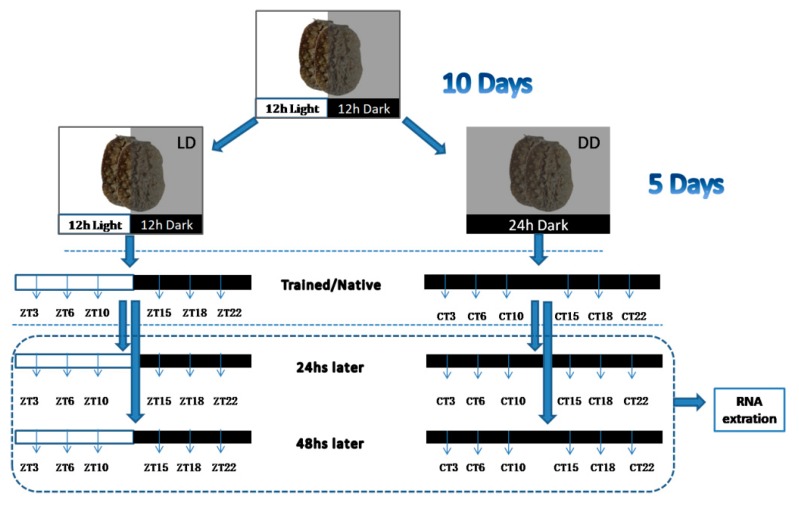
Experimental procedure for the whole experiment. A total of 216 animals were used in these experiments. Animals were trained at ZT/CT 3, 6, 10, 15, 18, and 22 after five-day treatment in an LD/DD cycle. After 24 and 48 h, all trained animals were tested at the same time points (ZT/CT 3, 6, 10, 15, 18, and 22). *N* = 6 for every time point in the behavioral experiment and *n* = 3 for every time point in the molecular experiment. ZT: zeitgeber time, CT: circadian time, LD: light–dark, DD: dark–dark.

Half of the sample was transferred from LD cycles into constant dark (DD) cycles for five days to test the effect of light–dark cycles (Figure 1). *Onchidium* photoreception is poor at wavelengths above 620 nm [34], and thus to facilitate observation for the experimenter, all experiments in darkness conditions were illuminated with a dim red light (light intensity was under 6 lux). Zeitgeber time (ZT, time-giver) is a notation for the time during an entrained circadian cycle; ZT = 0 corresponds to first light, which is 06:00 local time. The circadian time (CT) approximates to the ZT in DD.

### 2.2. Training Experiments

**Figure 2:**
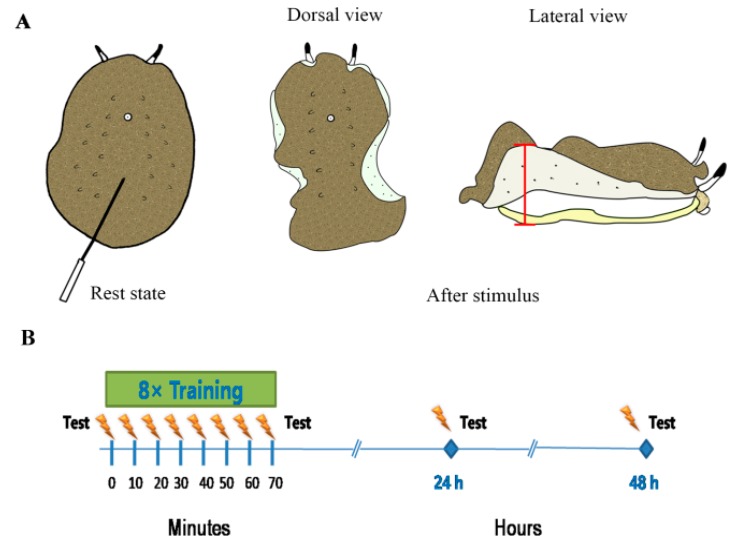
The mantle-upturned reflex training of *Onchidium reevesii* and experimental protocol for each animal. (**A**) Mantle-upturned reflex (MUR) of *O. reevesii*. When animals received the stimulus in the notum, their mantle elevated, as shown in the dorsal view and lateral view. The red line segment is the MUR amplitude. (**B**) Experimental protocols. The timing of training and testing is shown at relative intervals. The duration of the first-time shock was seen as the test result in “before training”, and the duration of the last shock in “8x training” was seen as the test result of acquisition. After 24 h and 48 h, consolidation testing was performed using one shock. The experimental lighting conditions depended on the cycles that those animals experienced.

The animals were measured for the sensitization of their mantle-upturned reflex (MUR) (Figure 2A). Gotow et al. [35] described the elevation movement; this response could be elicited by direct electrical stimulation in neurons. The amplitude and duration of elevation depended on the frequency of electrical stimulation. With increased frequency, the response to the stimuli became larger. When they received an electric shock, their mantle upturned and exposed the hyponotum (Figure 2). The animals were trained by electric shock while simultaneously measuring MUR duration. The sample was placed on its foot in the center of the table and allowed to acclimate for 6 min at rest; the mantle of the *Onchidium* was in a relaxed state, contrary to its risen state during simulation. After the stimulation, MUR duration began once the mantle was upturned (raised) and ended when the mantle was relaxed. The animal received training, which consisted of eight pulses of electricity (4 mA, 1 s in duration) delivered to the notum at 10-min intervals (Figure 2B). The position of shock was at ~2/3 of the notum (close to posterior end of notum). During the consolidation stage, the animals were returned to their environmental condition until the post-test at the same time point on the next day.

### 2.3. Cloning of cry1 and per2 Genes and Quantitative Analysis of OnCREB1, cry1, and per2 Genes

We sampled the ganglion during different circadian points under LD/DD conditions. Animals were anaesthetized with MgCl_2_ before dissection [36] and central nervous systems (CNSs) were immediately flash-frozen in liquid N_2_ before extraction. Total RNA was extracted from the tissues with RNAiso Plus (TaKaRa, Kusatsu, Japan) according to the manufacturer’s recommended protocol. Then, the absorbance at A_260 nm_/A_280 nm_ and A_230 nm_/A_260 nm_ was measured to determine the quality and purity of RNA. Complementary DNA (cDNA) was synthesized from the ganglion messenger RNA (mRNA) using a reverse transcription (RT) reagent kit with genomic DNA (gDNA) Eraser (TaKaRa) according to the manufacturer’s instructions. All primers were designed using Primer 5.0 software (PREMIER Biosoft International, Palo Alto, CA, USA). 

The 3′ and 5′ ends of the cDNA were obtained using the rapid amplification of cDNA ends (RACE) technique (TaKaRa). Partial fragments of *cry1* and *per2* genes were obtained from the de novo transcriptomic library. To confirm the fragment sequences, we used specific primers to amplify the partial fragments and re-sequenced the PCR products. The specific primers used for cloning the full-length cDNA of *per2* and *cry1* are provided in Table 1. The PCR cycling conditions were as follows; 94 °C for 5 min, followed by 30 cycles of 94 °C for 30 s, 58 °C for 30 s, and 72 °C for 1 min. The smart 5′-RACE (5′ Full RACE Kit, TaKaRa) and 3′-RACE kits (3′-Full RACE Core Set Ver. 2.0, Takara) were used according to the manufacturers’ instructions. The RACE-PCR product was ligated into pGEM-T Easy vector (Promega, Madison, WI, USA) and transformed into competent *E. coli* DH5-α cells. After blue-white selection and PCR identification, positive clones were selected and sequenced.

After obtaining the cDNA, the mRNA levels of *OnCREB1* (MK801136), *cry1* (MK801137), and *per2* (MK801138) were studied by fluorescent real-time (RT)-PCR. In our experiments, the 18S ribosomal RNA of *O. reevesii* was used as a housekeeping control gene [37], and 18S ribosomal RNA expressions maintained their stability in ganglion tissue over 24 h in our pre-experiment. Quantitative RT-PCR was performed using the Light Cycler^®^ 480 II instrument (Roche, Basel, Switzerland) with a QuantiFast^®^ SYBR^®^ Green PCR kit (Qiagen, Hilden, Germany). The qRT-PCR steps were as follows: 94 °C for 5 min, followed by 30 cycles of 94 °C for 30 s, 51 °C for 30 s, and 72 °C for 1 min, and a final step at 72 °C for 5 min. Data were collected from each qRT-PCR experiment performed in triplicate and expressed as the mean ± SEM (standard error of the mean). All the primers used in this process are listed in Table 1.

## 3. Results

### 3.1. Learning/Memory Acquisition and Long-Term Memory Expressed a Certain Rhythm in Different Conditions

**Figure 3:**
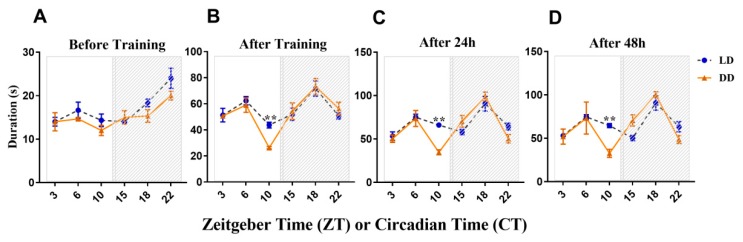
The rhythms of mantle-upturned reflex duration are shown for *Onchidium* examined in constant darkness (DD) (blue circles) and light–dark cycle (LD) (orange triangle) conditions. (**A**) The duration of the baseline mantle-upturned reflex before long-term memory (LTM) training shows an increased tendency of duration in the night-time. Moreover, no significant differences were observed in the duration of the mantle-upturned reflex between ZT and CT, and there was no significant effect of lighting condition × sampling time (*p* = 0.264). (**B**) Rhythm in the acquisition. Peak durations observed at CT/ZT 6 and CT/ZT 18. The duration in the “after training” stage revealed a significant effect of lighting condition (lighting condition, *p* < 0.05), as well as a significant effect of sampling time (time, *p* < 0.01). Bonferroni post hoc tests revealed that the durations between ZT 10 and CT 10 were significantly different (** *p* < 0.01). (**C**) A subset of animals was tested after 24 h to determine whether the rhythm in LTM persisted under LD/DD (lighting condition, *p* < 0.05; time, *p* < 0.001). The durations between ZT 10 and CT 10 also exhibited significant differences (** *p* < 0.01). (**D**) Animals were tested after 48 h to determine whether the rhythm in LTM persisted under LD/DD. There was no significant effect of lighting condition × sampling time (two-way analysis of variance (ANOVA), *p* > 0.05), whereas the duration at ZT 10 and CT 10 showed significant differences (** *p* < 0.01). Statistical analyses of all data were performed using two-way ANOVA with Bonferroni post hoc analysis for comparisons between groups. Values are means ± SEM (standard error of the mean) (*n* = 6 for ZT 3, 6, 10, 15, 18, and 22 and CT 3, 6, 10, 15, 18, and 22).

The difference in LTM might be driven by the light–dark cycle, so darkness was set as a constant to test the LD masking regulation. As the results show in Figure 3A, the duration of the baseline mantle-upturned reflex was not significantly different in animals measured in LD compared with DD. Moreover, the mantle-upturned reflex of *Onchidium* was less sensitive during the daytime than during the night-time in both LD and DD. The most sensitive period of time appeared late at night. During the daytime, the arousal threshold of *Onchidium* was elevated before training, which corresponds to the fact that *Onchidium* are nocturnal animals (Figure 3A). 

The training of *Onchidium* in ZT and CT had no significant effect on learning ability, except at ZT/CT 10 (Figure 3B). For animals trained at ZT 10, less sensitivity was acquired compared with that at CT 10, but this rather suggests that differences in observed learning abilities could be due to condition differences in the induction of the baseline mantle-upturned reflex (Figure 3A). Different rhythm patterns were observed before and after training, showing that the rhythm patterns in learning are controlled by the circadian rhythm. The learning pattern showed differences between ZT 10 and CT 10, suggesting that the LD cycles influence the learning in this time period.

The rhythms in LTM (Figure 3C,D) showed the same tendency as that in the animals’ initial learning (Figure 3B). These results further support the conclusion that the rhythm in LTM is not directly subject to the LD cycle. Moreover, the important reason for this rhythm shown in LTM is that the animals trained had different amounts of initial learning. Together, these results suggest that, most of the time, the rhythm pattern of LTM was due to the amount of initial learning. 

### 3.2. Expressions of cry1, per2, and OnCREB1 under The Control of Light–Dark (LD) and Constant Darkness (DD) Cycles

**Figure 4:**
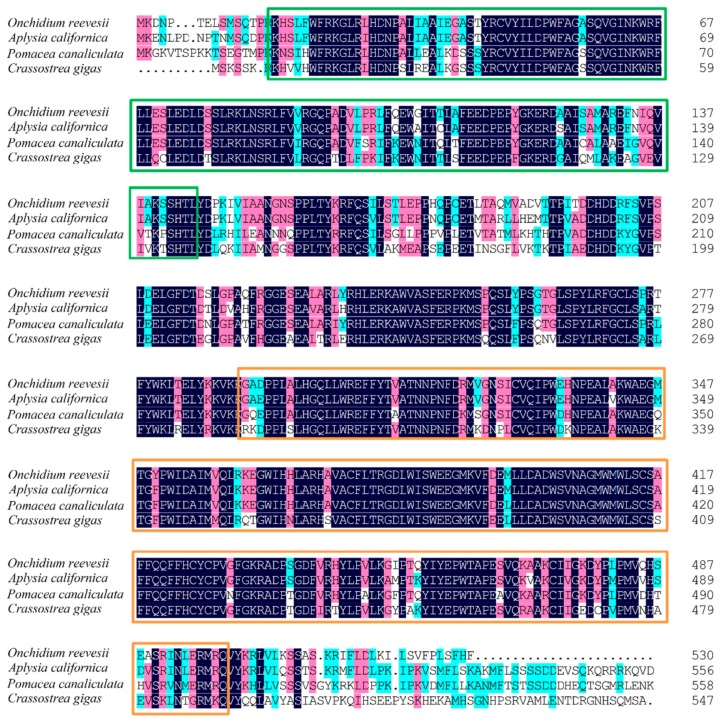
Alignment of conserved regions for CRY1. The *Onchidium* CRY1 protein contained conserved domains present in orthologs for Aplysia, Pomacea, and Crassostrea: green frame indicates the PHR domain and orange frame indicates the FAD binding domain. The blue shading indicates identical residue, pink shading indicates residues with strongly similar properties, and the cyan shading indicates residues with weakly similar properties.

**Figure 5:**
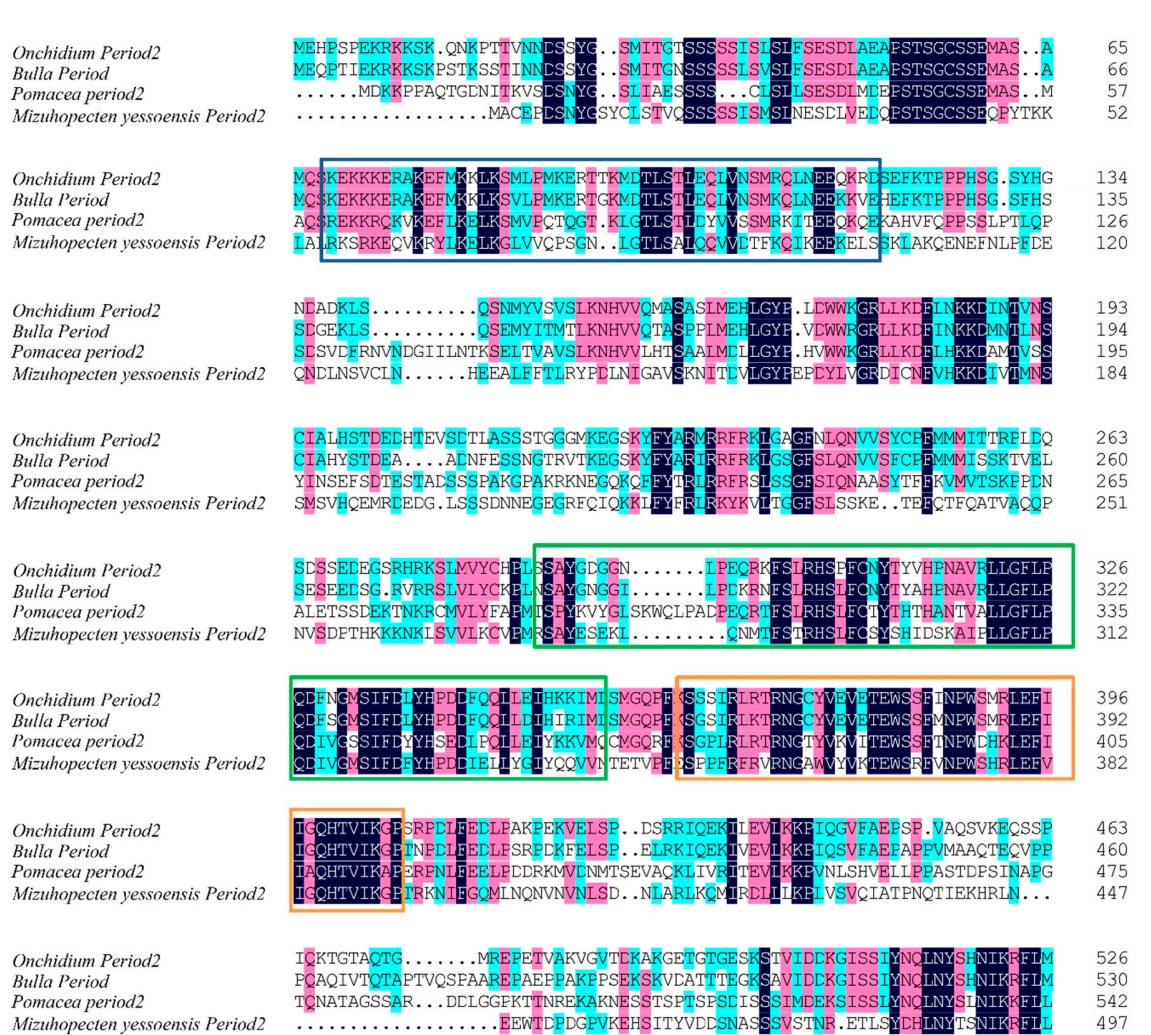
Alignment of conserved regions for PERIOD2. The *Onchidium* PERIOD2 protein contained conserved domains present in orthologs for Bulla, Pomacea, and Mizuhopecten: blue frame indicates the HLH domain, green frame indicates the PAS domain and orange frame indicates the PAC binding domain. The blue shading indicates identical residue, a pink shading indicated residue with strongly similar properties, and the cyan shading indicates residues with weakly similar properties.

We identified the *per2* and *cry1* genes of *Onchidium* (Figure 4, Figure 5, Figure S1 and Figure S2); the full length of *per2* is 7613 bp (MK801138) and *cry1* is 3131 bp (MK801137). The predicted *Onchidium* CRY1 protein contained the conserved regions present in orthologs of other species, including photolyase related domain (PHR domain) and flavin adenine dinucleotide binding domain (FAD binding domain) (Figure 4). The predicted *Onchidium* PERIOD2 protein contained the conserved regions present in orthologs of other species, including HLH, PAS, and PAC domains (Figure 5). All accession numbers for related proteins appear in Table S1.

**Figure 6:**
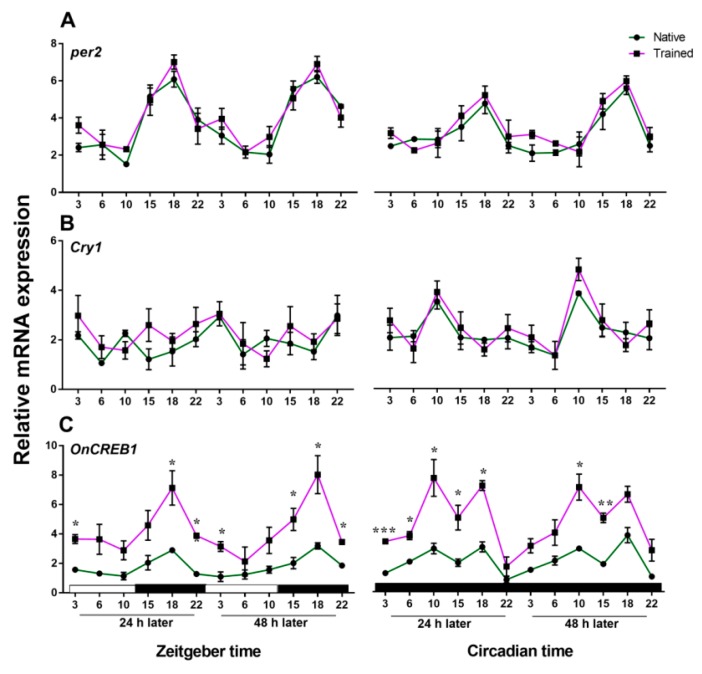
The expression comparison of related genes between native and trained group under LD and DD cycle. (**A**) Expression comparison of *per2*. Quantitative analysis of *per2* revealed that there was not significantly different between native and trained animals, and no interaction between both factors (sampling time × training condition) were detected (LD, *p* > 0.05; DD, *p* > 0.05). (**B**) Expression comparison of *cry1*. There was no significant effect of sampling time × training condition on *cry1* (LD, *p* > 0.05; DD, *p* > 0.05). (**C**) Expression comparison of *OnCREB1*. There was a significant effect of sampling time × training condition on *OnCREB1* expression (LD, *p* = 0.013; DD, *p* < 0.01). The training increased the *OnCREB1* expression, but not significant at all time points. * indicates *p* < 0.05; ** indicates *p* < 0.01; *** indicates *p* < 0.001. Statistical analyses of all data were performed using two-way analysis of variance (ANOVA) with Bonferroni post hoc analysis for comparisons between groups. Error bars in the figure represent SEM (*n* = 3 for every time point in each group).

For *OnCREB1*, *cry1*, and *per2* expressions were tested after 24 and 48 h following training and no training. As the transcriptional factor gene (*OnCREB1*) is important for memory consolidation, the transcriptional oscillation gene *per2* is crucial for the circadian clock and *cry1* is related to the circalunar/circatidal clock. The RNA dynamic expressions of the three genes were assessed next to determine whether those genes are rhythmic in the central nervous system. As an intertidal animal, the daily activity of *Onchidium* is synchronized not only by their circadian clock, but also by their circalunar/circatidal clock [38,39]. We tested the temporal expression pattern of *cry1*, *per2*, and *OnCREB1* throughout the daily cycle (CT/ZT 3, 6, 10, 15, 18, and 22). We also measured the expression of *per2*, *cry1*, and *OnCREB1* in the native group without any training. *Per2* and *cry1* expression levels were not significantly changed compared with those in the trained group (Figure 6A,B). Strikingly, the expression of *OnCREB1* in the trained group was increased by training (Figure 6C), and the expression of *OnCREB1* in the native group was similar to that in the trained group.

**Figure 7:**
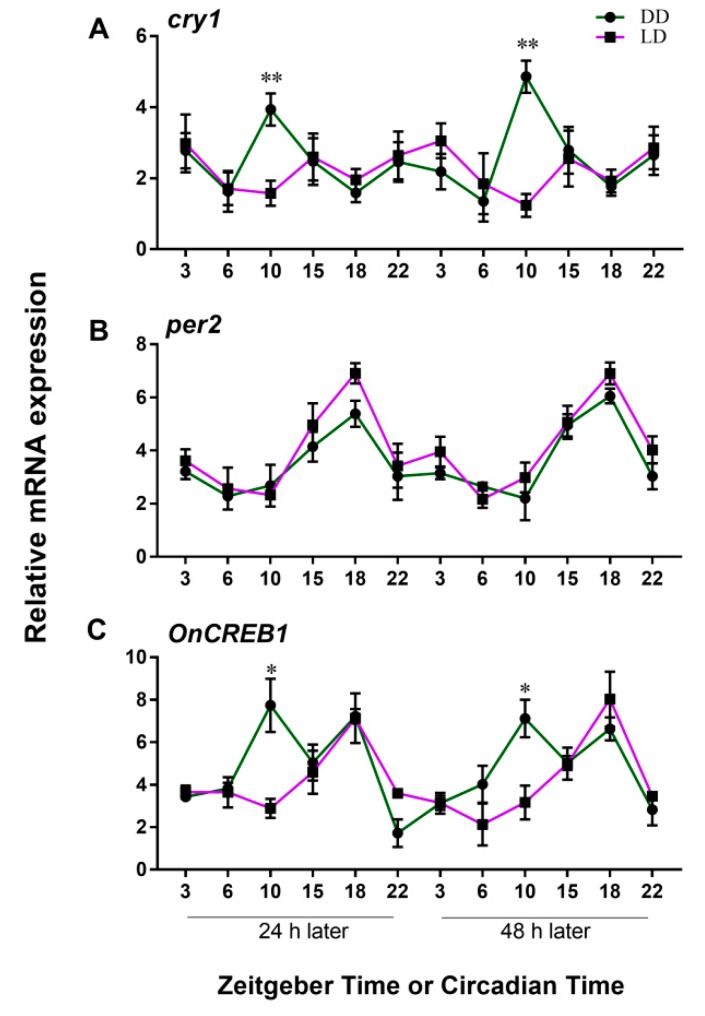
Temporal expression patterns of *cry1*, *per2*, and *OnCREB1* in *Onchidium reevesii* under LD and DD cycles. (**A**) Expression of *cry1*. The expression of *cry1* showed a significant effect based on lighting conditions (light, *p* < 0.05; time, *p* > 0.05). *Cry1* expressed irregularly in LD conditions (*cry1* LD, *p* > 0.05) and expressed rhythmically in DD conditions (*cry* DD, *p* < 0.05), with peak expression at CT 10 (Bonferroni’s post hoc analysis, ** *p* < 0.01 for ZT 10 vs. CT 10 at 24 and 48 h). (**B**) Expression of *per2*. Quantitative analysis of *per2* revealed a significant effect of sampling time (time, *p* < 0.05) but not lighting conditions (light, *p* > 0.05). The *per2* transcripts of LD and DD cycled in phase with each other, with a peak expression time point at CT/ZT 24. (**C**) Temporal profiles of *OnCREB1* expression sampled in LD and DD. There was a significant effect of lighting condition × sampling time on *OnCREB1* expression (*p* < 0.05). *OnCREB1* has two peak expressions at CT 10 and CT 18 under constant darkness, and one peak expression at ZT 18 under the light–dark cycle (Bonferroni post hoc analysis, * *p* < 0.05 for ZT 10 vs. CT 10 at 24 and 48 h). Statistical analyses of all data were performed using two-way ANOVA with Bonferroni post hoc analysis for comparisons between groups. Values are means ± SEM (*n* = 3 for ZT 3, 6, 10, 15, 18, 22 and CT 3, 6, 10, 15, 18, 22).

In trained animals, the peak expression of the *cry1* gene appeared at CT 10 under DD conditions, and the expressions under LD conditions were arrhythmic (two-way ANOVA, *p* > 0.05) (Figure 7A). For the *per2* gene, the expression level varied with circadian cycle, with peak expression being at ZT/CT 18 (Figure 7B). *OnCREB1* (in LD conditions) and *per2* transcripts cycled in phase with each other (Figure 7C). *OnCREB1* had two peak expression levels at ZT 10 and ZT 18. The *OnCREB1* transcript at CT 10 was in antiphase with the LTM, as shown in Figure 3C. The results show that the *OnCREB1* expressions are subject not only to circadian rhythm, but also the cycle change. CREB1 has an important transcriptional function in the pathway of long-term memory. As a consequence, the results also suggested that long-term memory formation is associated with circadian rhythm and the LD cycle.

## 4. Discussion

The intertidal mollusk *Onchidium reevesii* lives in the shoreline area between high and low tides. These organisms exhibit clocks which are not only tuned to day/night alterations, but also respond to high/low tide and moon phases. Some studies have suggested that memory is due to circadian regulation [40,41]. The present study described the learning and memory pattern of *Onchidium reevesii* under circadian modulation and LD cycle regulation.

Previous experiments have described the sleep behavioral characterizations to determine the invertebrate rest state [42]. The *Onchidium* rest state meets those criteria for sleep, and we found that *Onchidium* exhibited robust locomotion mainly during the night.

Interestingly, after training, *Onchidium* displayed a different duration pattern to that before training. The *Onchidium* show a time-of-day effect on memory acquisition, with one elevation during the light period at ZT 6, and another elevation during the dark period at ZT 18. Sometimes, entraining stimuli controlled by oscillators have direct effects on locomotors (e.g., the locomotor activity of crabs is influenced by light). This process is called “masking” [43,44]. To determine whether the memory acquisition was completely due to the endogenous rhythm system or masking, animals were trained in DD conditions and, surprisingly, showed a significant difference at CT 10 compared with ZT 10. The activity, like circatidal activity, might have been overridden by the LD cycle. Moreover, the masking of circatidal activity rhythm in intertidal animals is not unusual, and this phenomenon is shown in *Carcius maenas*, crabs, *Limulus polyphemus*, and *Apteronemobius asahinai* [2,3,45,46,47]. This masking mechanism makes animals adapt to changes in the environment [32]. This can explain the fluctuation at CT/ZT 10, the junction period between day and night, suggesting that the learning rhythm is regulated by changes in cycles and the endogenous rhythm system. 

Another study tested the circadian and diurnal effects on the LTM process (Figure 3C,D). However, the same tendency was previously observed, and the results for behavior suggested that LTM is largely subject to the amount of information/learning acquired initially. The results are difficult to square with other model species that involve different rhythmic modulations of learning and memory [38,48,49], suggesting not only the involvement of circadian regulations, but also other rhythms or phenomena. However, it is difficult to reach the conclusion that the LD cycle masks the circatidal rhythm, and the activity pattern is entirely subject to the circadian rhythm only through behavioral data. Therefore, molecular results in certain genes should be combined with behavioral data. 

*OnCREB1* gene expression after 24 and 48 h showed the same tendency as the *per2* gene during the light–dark (LD) cycle, which suggested that memory-related gene *OnCREB1* expression is associated with the circadian rhythm. Some studies showed that overexpressing the *per* gene in flies could enhance long-term memory [50]. The *per* gene encodes an essential component of the circadian clock, and *per* regulates the circadian process through a transcriptional negative feedback loop [51,52]. Furthermore, a previous study found that phosphorylation of MAPK underwent a circadian oscillation, and MAPK can activate the CREB [53,54]. Thus, the circadian rhythms in memory-related gene *OnCREB1* might also be related to the circadian gene *per2*. Constant darkness is necessary to evaluate whether the endogenous circadian rhythm drives the time-of-day effect, and it can also test other rhythms without the light–dark zeitgeber. Surprisingly, the *OnCREB1* gene had two peak expressions under the complete darkness condition (DD), the tendency of which was different from that in LD. Interestingly, a new peak was expressed rhythmically, the time point of which, in accordance with ZT 10, showed MUR differences in behavior experiments. Intertidal animals can release their previous rhythms (circalunar and circatidal rhythms) when exposed to the DD cycle [3,32]. Therefore, the critical agent in circatidal/circalunar rhythm is tested by the *cry1* gene. A previous study suggested that *cry1* is connected with behavioral tidal rhythms [10], and that *cry1* was sensitive to moonlight and likely to be controlled by a key element of the lunar clock [16]. The *cry* gene has been explored in previous research to study its role in the circatidal rhythm and its relationship with circalunar rhythm [10,16]. *Cry1* expressed irregularly in the LD cycle, and the steady expression peak of *cry1* shown at CT 10 in DD is the same as that of the time points of the peak expression of *OnCREB1*. *Cry1* increased at CT 10 under DD conditions but showed a decreasing sensitization response, consistent with *cry* expression, inhibiting the phosphorylation of CREB and thus influencing memory activity [55]. The increasing *OnCREB1* mRNA level at CT10 was opposite to that of memory activity, and similar negative feedback was also found in Aplysia [56]. The peak expression of *cry1* and *OnCREB1* at CT 10 was not shown in LD but appeared in DD, suggesting that these are circalunar rhythm-related genes and memory-related genes, at least partially suppressed by LD cycles. The *per2* expression pattern did not show differences between LD and DD, and it seemed that the endogenous circadian clock enables them to express a daily rhythm in the absence of light–dark cues. Therefore, the memory pattern in LD is possibly the result of an LD masking effect and circadian rhythm, and the memory activity during the DD cycle might be controlled by the interaction of circadian and other rhythms without the LD masking effect. Although the transcriptional regulation of *CREB1* genes was associated with long-term memory [57], the phosphorylation of CREB is also a critical step with implications on long-term memory [58]. Therefore, the phosphor-CREB/total CREB ratio could also be considered to enhance the study of LTM.

The study shows that the sea slug *Onchidium* harbors more than a circadian clock, even when kept in a laboratory for a long time. The results of this study were different from the rhythms of LTM in other model species [59,60]; this can be explained by the difference in species and species behavior. For instance, no circadian modulation in LTM has been observed in some rat species, while some studies show that strain differences affect circadian modulation in other rat species [61]. Another reason is that *Onchidium*, as an intertidal animal, lives in a harsh environment and thus harbors more biological rhythms [1,7], and the results shown in LTM contribute to the interactions of the circadian clock and LD cycle. Taken together, these results established relationships among LTM, circadian rhythms, and the LD cycle. A limitation of this study is the lack of monthly data for *cry1* expression and behavior experiments. We gathered those data every few hours more than four days apart, and the small sample size did not allow for a longer experiment. The free-running rhythm was not considered in this study and will also need to be pursued further to make the conclusion more complete. Further studies will be necessary to determine the LTM pattern of *Onchidium* under natural conditions over a long period to study circatidal modulation.

## Figures and Tables

**Table 1:** Polymerase chain reaction (PCR) primers used in gene cloning and realtime (RT)-PCR.

Usage	Primer Name	Primer Sequence (5′-3′)	Description
RT-PCR	Test-1F	GTTTAAAACGCCACCCCCAC	Used to amplify one part of the *per2* fragment
	Test-1R	ATGCTGAACTGAGTGGGTGG	
	Test-2F	TTCGAAAACTGGGTGCAGGT	Used to amplify one part of the *per2* fragment
	Test-2R	GTCTCAGGCTCTCTCATGCC	
	Test-3F	ATGAGAGAGCCTGAGACCGT	Used to amplify one part of the *per2* fragment
	Test-3R	TGCGCATGTTCAGAAGGAGT	
	Test-4F	TCGTGAATCAGCGGACAACA	Used to amplify one part of the *per2* fragment
	Test-4R	GAGGGCGATAGAAGCCAGTC	
	Test-5F	ACATCCCACCAGAAACGTCC	Used to amplify one part of the *per2* fragment
	Test-5R	ATGGGCTCCTGAAACATGGG	
	Test-6F	CAGGTGATGACTGGCTTCTATC	Used to amplify one part of the *per2* fragment
	Test-6R	CTGGTGTGTCGTAGTTCTCATC	
	Test-7F	CCCTAAGGATGGGTCCCTCA	Used to amplify one part of the *per2* fragment
	Test-7R	CACCGTGGCTGTTGTTGATG	
	Test-8F	AAACCCTCACTGCCCAGATG	Used to amplify one part of the *cry1* fragment
	Test-8R	GTGAGAAAGCAAGCCACAGC	
	Test-9F	GTGTACAAGAGGCTGGTGCT	Used to amplify one part of the *cry1* fragment
	Test-9R	AAAAGCCCTCAGGAGCTGAC	
	Test-10F	GTCAGCTCCTGAGGGCTTTT	Used to amplify one part of the *cry1* fragment
	Test-10R	CCTTGTCTGCGTCACAAAGC	
	Test-11F	GGTGCCTCACAAGTAGGAATTA	Used to amplify one part of the *cry1* fragment
	Test-11R	GATTGTGTTCCCACGGTATCT	
	Test-12F	GATGCTGACTGGAGCGTAAA	Used to amplify one part of the *cry1* fragment
	Test-12R	CCCTCAGGAGCTGACTAATAAAC	
	Test-13F	AGACTGCAGACCCTGTTAATG	Used to amplify one part of the *cry1* fragment
	Test-13R	ATGGGCTAGAGAGCTGATACT	
	Test-14F	ATGGAAACAGCCCACCACTC	Used to amplify one part of the *cry1* fragment
	Test-14R	TCCACCACGAAACTGAGCTG	
	Test-15F	AGCTCAGTTTCGTGGTGGAG	Used to amplify one part of the *cry1* fragment
	Test-15R	GGGGATAGTCCTTGCCGATG	
RACE	3′RACE-F1	CCCTAAGGATGGGTCCCTCA	Gene-specific outer primer for *per2*
	3′RACE-F2	AATGTGTCTCTCAGCCCTGC	Gene-specific inner primer for *per2*
	3′RACE-F3	GTCAGCTCCTGAGGGCTTTT	Gene-specific outer primer for *cry1*
	3′RACE-F4	AGACTGCAGACCCTGTTAATG	Gene-specific inner primer for *cry1*
	3′RACE outer primer	TACCGTCGTTCCACTAGTGATTT	Primers from kit
	3′RACE inner primer	CGCGGATCCTCCACTAGTGATTTCACTATAGG	
	5′RACE-R1	ATGCTGAACTGAGTGGGTGG	Gene-specific outer primer for *per2*
	5′RACE-R2	AGACGAATGCTGCTGCTCTT	Gene-specific inner primer for *per2*
	5′RACE-R3	GATTGTGTTCCCACGGTATCT	Gene-specific outer primer for *cry1*
	5′RACE-R4	TCCACCACGAAACTGAGCTG	Gene-specific inner primer for *cry1*
	5′RACE outer primer	CATGGCTACATGCTGACAGCCTA	Primers from kit
	5′RACE inner primer	CGCGGATCCACAGCCTACTGATGATCAGTCGATG	
qRT-PCR	qRT-PCR primer F	TGTTGAGTCCGCCAACCTTT	Used to amplify the *per2* fragment for real-time PCR
	qRT-PCR primer R	AGTGGCTGCTCCTCTGAAAC	
	qRT-PCR primer F	GATGCTGACTGGAGCGTAAA	Used to amplify the *Cry1* fragment for real-time PCR
	qRT-PCR primer R	CCAAAGCCCACAGGACAATA	
	qRT-PCR primer F	CCAGTTGGAGGAACCAATGT	Used to amplify the *OnCREB1* fragment for real-time PCR
	qRT-PCR primer R	CATGTGCTGTGGACTTGAAATAG	
	18S primer F	TCCGCAGGAGTTGCTTCGAT	Used to amplify the 18S fragment for real-time PCR
	18S primer R	ATTAAGCCGCAGGCTCCACT	

## Supplementary Materials

The following are available online at https://www.mdpi.com/2073-4425/10/7/488/s1; Figure S1: Phylogenetic tree of CRY family proteins, Figure S2: Phylogenetic tree of PERIOD family proteins, Table S1: Accession numbers for proteins.

## References

[1] Arey L.B., Crozier W.J. (1918). The ‘homing habits’ of the pulmonate mollusk *Onchidium*. Proc. Natl. Acad. Sci. USA.

[2] Naylor E. (1958). Tidal and diurnal rhythms of locomotory activity in *Carcinus maenas* (L.). J. Exp. Biol..

[3] Dubofsky E.A., Simpson S.D., Chabot C.C. (2013). Patterns of activity expressed by juvenile horseshoe crabs. Biol. Bull..

[4] Forward R.B., Diaz H., Cohen J.H. (2005). The tidal rhythm in activity of the mole crab *Emerita talpoida*. J. Mar. Biol. Assoc. UK.

[5] Barker G.M. (2001). Biology of Terrestrial Molluscs.

[6] Naylor E. (2010). Chronobiology of Marine Organisms.

[7] Kristin T.R., Florian R., Enrique A. (2015). Another place, another timer: Marine species and the rhythms of life. Bioessays News Rev. Mol. Cell. Dev. Biol..

[8] Deborah B.P., Cassone V.M., Earnest D.J., Golden S.S., Hardin P.E., Thomas T.L., Zoran M.J. (2005). Circadian rhythms from multiple oscillators: Lessons from diverse organisms. Nat. Rev. Genet..

[9] Hardin P.E., Hall J.C., Rosbash M. (1990). Feedback of the *Drosophila* period gene product on circadian cycling of its messenger RNA levels. Nature.

[10] Mat A.M., Perrigault M., Massabuau J.C., Tran D. (2016). Role and expression of *cry1* in the adductor muscle of the oyster *Crassostrea gigas* during daily and tidal valve activity rhythms. Chronobiol. Int..

[11] Tei H., Okamura H., Shigeyoshi Y., Fukuhara C., Ozawa R., Hirose M., Sakaki Y. (1997). *Circadian oscillation* of a mammalian homologue of the *Drosophila* period gene. Nature.

[12] Padmanabhan K., Weitz C.J. (2012). Feedback regulation of transcriptional termination by the mammalian circadian clock PERIOD complex. Science.

[13] Cyran S.A., Buchsbaum A.M., Reddy K.L., Meng-Chi L., Glossop N.R.J., Hardin P.E., Young M.W., Storti R.V., Justin B. (2003). *vrille*, *Pdp1*, and *dClock* form a second feedback loop in the *Drosophila circadian* clock. Cell.

[14] Levy O., Appelbaum L., Leggat W., Gothlif Y., Hayward D.C., Miller D.J., Hoegh-Guldberg O. (2007). Light-responsive cryptochromes from a simple multicellular animal, the coral *Acropora millepora*. Science.

[15] Todo T., Ryo H., Yamamoto K., Toh H., Inui T., Ayaki H., Nomura T., Ikenaga M. (1996). Similarity among the *Drosophila* (6-4)photolyase, a human photolyase homolog, and the DNA photolyase-blue-light photoreceptor family. Science.

[16] Masato F., Takahiro T., Yuki T., Sung-Pyo H., Nozomi S., Akihiro T., Yoko K., Keiko O., Toshiyuki O. (2011). Lunar phase-dependent expression of cryptochrome and a photoperiodic mechanism for lunar phase-recognition in a reef fish, goldlined spinefoot. PLoS ONE.

[17] Feng D., Lazar M.A. (2012). Clocks, metabolism, and the epigenome. Mol. Cell.

[18] Dipesh C., Christopher S.C. (2002). Circadian modulation of learning and memory in fear-conditioned mice. Behav. Brain Res..

[19] Smarr B.L., Jennings K.J., Driscoll J.R., Kriegsfeld L.J. (2014). A time to remember: The role of circadian clocks in learning and memory. Behav. Neurosci..

[20] Dash P.K., Hochner B., Kandel E.R. (1990). Injection of the cAMP-responsive element into the nucleus of *Aplysia* sensory neurons blocks long-term facilitation. Nature.

[21] Bourtchuladze R., Frenguelli B., Blendy J., Cioffi D., Schutz G., Silva A.J. (1994). Deficient long-term memory in mice with a targeted mutation of the cAMP-responsive element-binding protein. Cell.

[22] Silva A.J., Kogan J.H., And P.W.F., Kida S. (1998). CREB and Memory. Annu. Rev. Neurosci..

[23] Gau D., Lemberger T., Gall C.V., Kretz O., Minh N.L., Gass P., Schmid W., Schibler U., Korf H.W., Schütz G. (2002). Phosphorylation of CREB Ser142 regulates light-induced phase shifts of the circadian clock. Neuron.

[24] Reppert S.M., Weaver D.R. (2002). Coordination of circadian timing in mammals. Nature.

[25] Mohamed H.A., Yao W., Fioravante D., Smolen P.D., Byrne J.H. (2005). cAMP-response elements in *Aplysia creb1*, *creb2*, and *Ap-uch* promoters: Implications for feedback loops modulating long term memory. J. Biol. Chem..

[26] Bartsch D., Casadio A., Karl K.A., Serodio P., Kandel E.R. (1998). *CREB1* encodes a nuclear activator, a repressor, and a cytoplasmic modulator that form a regulatory unit critical for long-term facilitation. Cell.

[27] Rawashdeh O., Jilg A., Maronde E., Fahrenkrug J., Stehle J.H. (2016). *Period1* gates the circadian modulation of memory-relevant signaling in mouse hippocampus by regulating the nuclear shuttling of the CREB kinase pP90RSK. J. Neurochem..

[28] Campos L.M., Cruz-Rizzolo R.J., Pinato L. (2015). The primate seahorse rhythm. Brain Res..

[29] Fernandez R.I., Lyons L.C., Jonathan L., Omar K., Arnold E. (2003). Circadian modulation of long-term sensitization in *Aplysia*. Proc. Natl. Acad. Sci. USA.

[30] Mcdonald R.J., Hong N.S., Ray C., Ralph M.R. (2002). No time of day modulation or time stamp on multiple memory tasks in rats. Learn. Motiv..

[31] Chabot C.C., Watson W.H. (2014). Daily and Tidal Rhythms in Intertidal Marine Invertebrates.

[32] Page T.L. (1989). Masking in invertebrates. Chronobiol. Int..

[33] Shen H., Li K., Chen H., Chen X., He Y., Shi Z. (2011). Experimental ecology and hibernation of *Onchidium struma* (Gastropoda: Pulmonata: Systellommatophora). J. Exp. Mar. Biol. Ecol..

[34] Nishi T., Gotow T. (1992). A neural mechanism for processing colour information in molluscan extra-ocular photoreceptors. J. Exp. Biol..

[35] Gotow T., Tateda H., Kuwabara M. (1973). The function of photoexcitive neurones in the central ganglia for behavioral activity of the marine mollusc, *Onchidium verruculatum*. J. Comp. Physiol..

[36] Shimotsu K., Nishi T., Nakagawa S., Gotow T. (2010). A new role for photoresponsive neurons called simple photoreceptors in the sea slug *Onchidium verruculatum*: Potentiation of synaptic transmission and motor response. Comp. Biochem. Physiol. Part A Mol. Integr. Physiol..

[37] Xu G., Yang T., Wang D., Li J., Liu X., Wu X., Shen H. (2018). A comprehensive comparison of four species of *Onchidiidae* provides insights on the morphological and molecular adaptations of invertebrates from shallow seas to wetlands. PLoS ONE.

[38] Zantke J., Ishikawa-Fujiwara T., Arboleda E., Lohs C., Schipany K., Hallay N., Straw A., Todo T., Tessmar-Raible K. (2013). Circadian and circalunar clock interactions in a marine annelid. Cell Rep..

[39] Goto S.G., Takekata H. (2015). Circatidal rhythm and the veiled clockwork. Curr. Opin. Insect Sci..

[40] Page T.L. (2015). Circadian regulation of learning and memory. Curr. Opin. Insect Sci..

[41] Garren M.V., Sexauer S.B., Page T.L. (2013). Effect of circadian phase on memory acquisition and recall: Operant conditioning vs. classical conditioning. PLoS ONE.

[42] Vorster A.P.A., Krishnan H.C., Chiara C., Lyons L.C. (2014). Characterization of sleep in *Aplysia californica*. Sleep.

[43] Marques M.D., Waterhouse J.M. (1994). Masking and the evolution of circadian rhythmicity. Chronobiol. Int..

[44] Rietveld W.J., Minors D.S., Waterhouse J.M. (1993). Circadian rhythms and masking: An overview. Chronobiol. Int..

[45] Palmer J.D. (1990). The rhythmic lives of crabs. Bioscience.

[46] Chabot C.C., Skinner S.J., Watson W.H. (2008). Rhythms of locomotion expressed by *Limulus polyphemus*, the American horseshoe crab: I. synchronization by artificial tides. Biol. Bull..

[47] Takekata H., Goto S.G., Satoh A., Numata H. (2014). Light masking of the circatidal activity rhythm in the mangrove cricket *Apteronemobius asahinai*. Biol. Rhythm Res..

[48] Wang M.C., Dragich J.M., Kudo T., Odom I.H., Welsh D.K., O’Dell T.J., Colwell C.S. (2009). Expression of the circadian clock gene *Period2* in the hippocampus: Possible implications for synaptic plasticity and learned behaviour. ASN Neuro.

[49] Bjorn R., Jan B. (2013). About sleep’s role in memory. Physiol. Rev..

[50] Takaomi S., Takuya T., Toshihiro K., Yoshiaki K. (2004). A clock gene, period, plays a key role in long-term memory formation in *Drosophila*. Proc. Natl. Acad. Sci. USA.

[51] Buhr E.D., Takahashi J.S. (2013). Molecular Components of the Mammalian Circadian Clock. Handbook of Experimental Pharmacology.

[52] Montenegro-Montero A., Canessa P., Larrondo L.F. (2015). Chapter Four—Around the Fungal Clock: Recent Advances in the Molecular Study of Circadian Clocks in *Neurospora* and Other Fungi. Adv. Genet..

[53] Mahan K.L. (2008). Circadian Oscillation of MAPK Activity and cAMP in the Hippocampus: Implications for Memory Persistence. Ph.D. Thesis.

[54] Adams J.P., Roberson E.D., English J.D., Selcher J.C., Sweatt J.D. (2000). MAPK regulation of gene expression in the central nervous system. Acta Neurobiol. Exp..

[55] Zhang E.E., Liu Y., Dentin R., Pongsawakul P.Y., Liu A.C., Hirota T., Nusinow D.A., Sun X., Landais S., Kodama Y. (2010). Cryptochrome mediates circadian regulation of cAMP signaling and hepatic gluconeogenesis. Nat. Med..

[56] Liu R.Y., Shah S., Cleary L.J., Byrne J.H. (2011). Serotonin- and training-induced dynamic regulation of CREB2 in *Aplysia*. Learn. Mem..

[57] Sadamoto H., Kitahashi T., Fujito Y., Ito E. (2010). Learning-dependent gene expression of CREB1 isoforms in the molluscan brain. Front. Behav. Neurosci..

[58] Mizuno M., Yamada K., Maekawa N., Saito K., Seishima M., Nabeshima T. (2002). CREB phosphorylation as a molecular marker of memory processing in the hippocampus for spatial learning. Behav. Brain Res..

[59] Lyons L.C., Oliver R., Ayelet K., Susswein A.J., Arnold E. (2005). Circadian modulation of complex learning in diurnal and nocturnal *Aplysia*. Proc. Natl. Acad. Sci. USA.

[60] Chiang C.K., Xu B., Mehta N., Mayne J., Sun W.Y.L., Cheng K., Ning Z., Dong J., Zou H., Cheng H.Y.M. (2017). Phosphoproteome profiling reveals circadian clock regulation of posttranslational modifications in the murine hippocampus. Front. Neurol..

[61] Cain S.W., Ko C.H., Chalmers J.A., Ralph M.R. (2004). Time of day modulation of conditioned place preference in rats depends on the strain of rat used. Neurobiol. Learn. Mem..

