# Supplementary material for: Effect of Circadian Clock and Light–Dark Cycles in Onchidium reevesii: Possible Implications for Long-Term Memory
Source: Genes (Basel). 2019 Jun 27;10(7):488. doi: 10.3390/genes10070488 (PMC6679201; doi:10.3390/genes10070488)
Supplement: Supplementary file 1 [file genes-10-00488-s001.pdf]

Supplementary Results

We identified a single *cry* gene in *Onchidium*. Phylogenetic analysis showed that it can be classified CRY1 group (Figure S1), which led us to identify it as *Onchidium* CRY1 (MK801137), and named the gene as *cry1*.

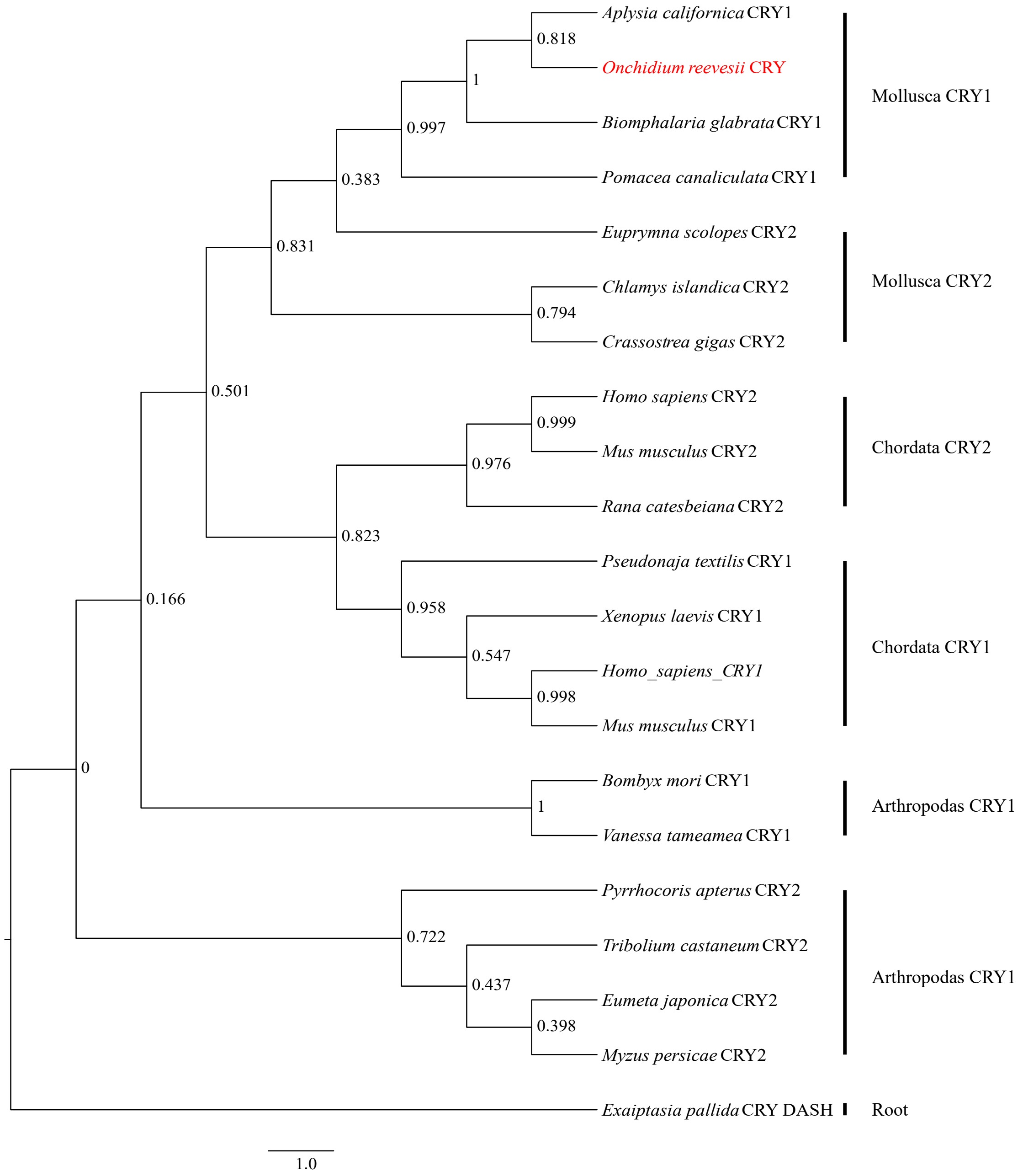

Figure S1 Phylogenetic tree of CRY family proteins.
Onchidium CRY sequences (CRY1 in present study) and other related sequences obtained from NCBI database (accession nos. are shown in Table S1) were analyzed using the maximum likelihood method (The number of bootstrap replications is 1000). The putative *Onchidium* CRY was closely related to orthologs in Mollusca CRY1 group. The tree was rooted on CRY DASH from the *Exaiptasia pallida*. The numbers at branches indicate the bootstrap values. Accession numbers for these proteins can be found in Table S1.

A single transcript for period gene was identified in *Onchidium*. Phylogenetic analysis showed that it can be classified PREIOD2 group (Figure S3), which led us to identify it as *Onchidium* PERIOD2 (MK801138).

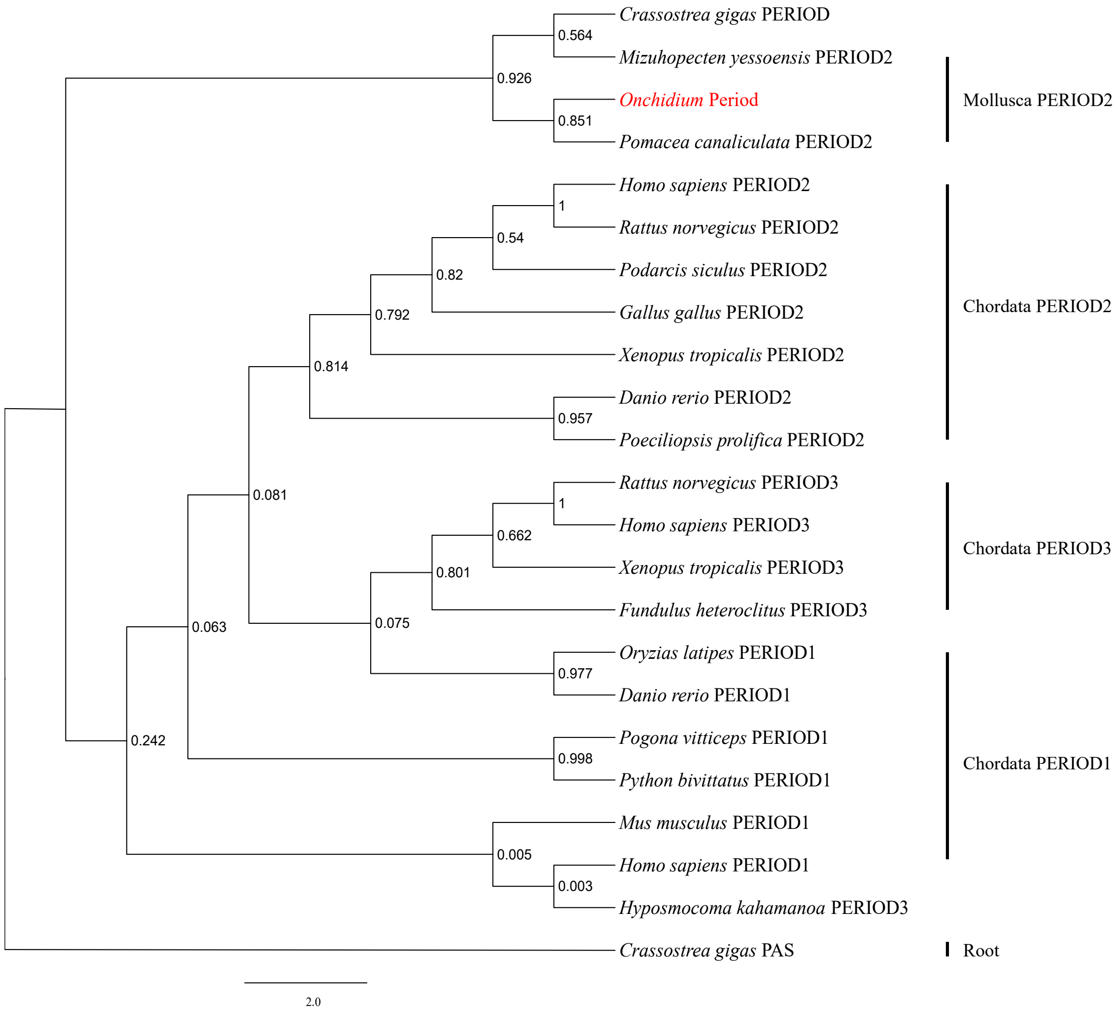

Figure S2 Phylogenetic tree of PERIOD family proteins. *Onchidium* PERIOD sequences (PERIOD2 in present study) and other related sequences obtained from NCBI database (accession nos. are shown in Table S1) were analyzed using the maximum likelihood method (The number of bootstrap replications is 1000). The putative *Onchidium* PERIOD was closely related to orthologs in Mollusca PERIOD2 group. The tree was rooted on PAS DOMAIN PROTEIN (PAS) from the oyster *Crassostrea gigas*. The numbers at branches indicate the bootstrap values. Accession numbers for these proteins can be found in Table S1.

Table S1 Accession numbers for related proteins

Accession Number	Description	Species	Protein
XP_012941094.1	PREDICTED: cryptochrome-1-like	*Aplysia californica*	*Aplysia californica* CRY1
XP_025096987.1	cryptochrome-1-like	*Pomacea canaliculata*	*Pomacea canaliculata* CRY1
XP_013080710.1	PREDICTED: cryptochrome-1-like	*Biomphalaria glabrata*	*Biomphalaria glabrata* CRY1
XP_026551073.1	cryptochrome-1	*Pseudonaja textilis*	*Pseudonaja textilis* CRY1
AAD39548.1	cryptochrome 1	*Mus musculus*	*Mus musculus* CRY1
NP_004066.1	cryptochrome-1	*Homo sapiens*	*Homo sapiens* CRY1
AAK94665.1	cryptochrome 1	*Xenopus laevis*	*Xenopus laevis* CRY1
NP_001182628.1	cryptochrome 1	*Bombyx mori*	*Bombyx mori* CRY1
XP_026498971.1	cryptochrome-1	*Vanessa tameamea*	*Vanessa tameamea* CRY1
GBP87169.1	Cryptochrome-1	*Eumeta japonica*	*Eumeta japonica* CRY1
AYE92099.1	Cryptochrome 2	*Chlamys islandica*	*Chlamys islandica* CRY2
EFA04537.1	cryptochrome 2	*Tribolium castaneum*	*Tribolium castaneum* CRY2
AAP13561.1	cryptochrome 2	*Rana catesbeiana*	*Rana catesbeiana* CRY2
AGI17567.1	cryptochrome 2	*Pyrrhocoris apterus*	*Pyrrhocoris apterus* CRY2
AUN43314.1	cryptochrome 2	*Myzus persicae*	*Myzus persicae* CRY2
AAD46561.1	cryptochrome 2	*Mus musculus*	*Mus musculus* CRY2
NP_001120929.1	cryptochrome-2	*Homo sapiens*	*Homo sapiens* CRY2
AGJ94015.1	cryptochrome-2	*Euprymna scolopes*	*Euprymna scolopes* CRY2
AQM57602.1	cryptochrome 2	*Crassostrea gigas*	*Crassostrea gigas* CRY2
XP_020903321.1	cryptochrome DASH	*Exaiptasia pallida*	*Exaiptasia pallida* CRY DASH
AQM57604.1	period circadian protein	*Crassostrea gigas*	*Crassostrea gigas* PERIOD
XP_025030999.1	period circadian protein homolog 1	*Python bivittatus*	*Python bivittatus* PERIOD1
XP_020666128.1	period circadian protein homolog 1	*Pogona vitticeps*	*Pogona vitticeps* PERIOD1
NP_001129992.1	period circadian protein homolog 1	*Oryzias latipes*	*Oryzias latipes* PERIOD1
NP_001152839.1	period circadian protein homolog 1	*Mus musculus*	*Mus musculus* PERIOD1
NP_002607.2	period circadian protein homolog 1	*Homo sapiens*	*Homo sapiens* PERIOD1
NP_997604.2	period circadian protein homolog 1	*Danio rerio*	*Danio rerio* PERIOD1
AAI66199.1	per2 protein	*Xenopus tropicalis*	*Xenopus tropicalis* PERIOD2
NP_113866.1	period circadian protein homolog 2	*Rattus norvegicus*	*Rattus norvegicus* PERIOD2
CAI43981.1	PER2 protein	*Podarcis siculus*	*Podarcis siculus* PERIOD2
XP_025083583.1	period circadian protein homolog 2-like	*Pomacea canaliculata*	*Pomacea canaliculata* PERIOD2
JAO88064.1	PER2	*Poeciliopsis prolifica*	*Poeciliopsis prolifica* PERIOD2
XP_021375509.1	period circadian protein homolog 2-like isoform X2	*Mizuhopecten yessoensis*	*Mizuhopecten yessoensis* PERIOD2
NP_073728.1	period circadian protein homolog 2	*Homo sapiens*	*Homo sapiens* PERIOD2
AAL98705.1	PERIOD2	*Gallus gallus*	*Gallus gallus* PERIOD2
NP_878277.2	period circadian protein homolog 2	*Danio rerio*	*Danio rerio* PERIOD2
NP_001072696.1	period circadian protein homolog 3	*Xenopus tropicalis*	*Xenopus tropicalis* PERIOD3
NP_076468.2	period circadian protein homolog 3	*Rattus norvegicus*	*Rattus norvegicus* PERIOD3
XP_026318965.1	period circadian protein homolog 3 isoform X1	*Hyposmocoma kahamanoa*	*Hyposmocoma kahamanoa* PERIOD3
NP_001276792.1	period circadian protein homolog 3 isoform 3	*Homo sapiens*	*Homo sapiens* PERIOD3
XP_021178406.1	period circadian protein homolog 3	*Fundulus heteroclitus*	*Fundulus heteroclitus* PERIOD3
EKC18855.1	Neuronal PAS domain-containing protein 4	*Crassostrea gigas*	*Crassostrea gigas* PAS
